# Case Report: Migralepsy: The Two-Faced Janus of Neurology

**DOI:** 10.3389/fneur.2021.711858

**Published:** 2021-10-11

**Authors:** Giorgia Sforza, Claudia Ruscitto, Romina Moavero, Fabiana Ursitti, Michela Ada Noris Ferilli, Samuela Tarantino, Martina Balestri, Federico Vigevano, Massimiliano Valeriani, Laura Papetti

**Affiliations:** ^1^Pediatric Headache Center, Department of Neuroscience, Bambino Gesù Children Hospital, Istituto di Ricovero e Cura a Carattere Scientifico, Rome, Italy; ^2^Child Neurology and Psychiatry Unit, Tor Vergata University of Rome, Rome, Italy; ^3^Child Neurology Unit, Bambino Gesù Children's Hospital, Istituto di Ricovero e Cura a Carattere Scientifico, Rome, Italy; ^4^Center for Sensory-Motor Interaction, Aalborg University, Aalborg, Denmark

**Keywords:** migralepsy, migraine, epilepsy, seizure, aura

## Abstract

We report three cases of pediatric patients suffering from migraine aura triggered seizures. This entity, also called migralepsy, still does not have a unique definition today. Migraine and epilepsy are both episodic neurological disorders with periods of interictal well-being; this is indicative of similar pathophysiological mechanisms, such as increased neuronal excitation and ion channel dysfunction. The purpose of this paper is to discuss the clinical and instrumental features of migralepsy through the description of three clinical cases in which the symptoms of the usual migraine aura developed into a generalized tonic–clonic or focal seizure.

## Introduction

Migraine is the most common type of primary headache in pediatric population and the prevalence varies according to presenting age, going from 3% in younger children to ~20% in adolescents (1, 2). Pediatric epilepsy is another frequent neurological condition, with a prevalence of approximately 3.2–5.5/1,000 in developed countries (3). There is a large range of comorbidity between migraine and epilepsy: among patients with migraine, epilepsy can occur in 1–17% of the cases (as in the healthy pediatric population) while, among epileptic patients, the prevalence of migraine ranges from 7 to 26% (4).

The International Headache Society (IHS) in the International Classification of Headache Disorders (ICHD-3 edition) identified three main associations between headache and epilepsy, including migraine-triggered seizure (migralepsy), hemicrania epileptica, and postictal headache (5).

Migralepsy is defined as a seizure triggered by a migraine attack with aura: in particular, the seizure must fulfill the diagnostic criteria for a specific seizure, it has to occur in a patient suffering from migraine with aura (MA), during, or within 1 h of a migraine attack with aura (5). To date several cases of patients with migralepsy have been described, many of which have turned out to be cases of occipital epilepsy (6, 7).

Therefore, the purpose of this study is to discuss the clinical and instrumental features of migralepsy through the description of three clinical cases in which the symptoms of the usual migraine aura developed into a generalized tonic–clonic or focal seizure.

## Case Series

Clinical characteristics of our patients are reported in Table 1. The parents of the patients signed informed consent for the publication of the data. Furthermore, the submission of the paper was validated by the ethics committee of the Bambino Gesù Hospital.

**Table 1:** Main features of clinical cases and Visual Aura Rating Scale (VARS).

	**Patient 1**	**Patient 2**	**Patient 3**
Gender	Female	Female	Female
Migraine age onset	7	5	8
Seizure age onset	11	6	12
**Visual Aura Rating Scale (VARS):**			
Duration 5–60 min (three points)	Yes	Yes	Yes
Temporal development ≥5 min (two points)	Yes	Yes	Yes
Scotoma (two points)	Yes	Yes	Yes
Fortification (two points)	No	No	No
Homonymus character (one point)	No	No	No
Brain MRI findings	None	None	None
Treatment	Topiramate 2 mg/kg/day	Valproic acid 20 mg/kg/day, replaced for side effects and poor efficacy with Topiramate 2 mg/kg/day	Topiramate 2 mg/kg/day
Outcome	Migraine	Migraine	Migraine/Isolated visual aura

### Patient 1

An 11-year-old girl was followed at our headache center, suffering from MA and without aura (MO) since the age of 7 according to the ICHD-3 criteria (5). She was born full term with a normal delivery and had normal developmental milestones. Her father suffered from MO, but no family member had a history of epilepsy.

When she was 7 years old, she developed MO attacks, which occurred once a week. Three years later, she began experiencing MA attacks. Her aura was characterized by the appearance of a black circle in the center of the visual field, which subsequently became a scintillating scotoma. These symptoms occurred for about 5–10 min, with the subsequent onset of a headache that persisted for a few hours. The headache had migraine characteristics with throbbing pain contralateral to the site of the visited aura and associated with photophobia, phonophobia, and vomiting. These attacks occurred two or three times a month, almost exclusively in the evening after studying.

At age 11, she presented two episodes characterized by her usual visual aura followed by a loss of consciousness, staring, falling to the ground, and subsequent evolution into tonic–clonic seizures.

The patient was hospitalized, and she underwent a magnetic resonance imaging (MRI) of the brain that was normal. An interictal EEG was also performed showing bilateral temporal occipital epileptic spike anomalies and photo paroxysmal response.

The girl reported that the visual aura that preceded the seizures was the same as the aura that used to precede her migraine attacks in the past. Long-term therapy with topiramate was initiated at a dose of 2 mg/kg/day. Control interictal-EEG performed 1 month after the beginning of the therapy did not show anomalies, but only photo paroxysmal response. She was diagnosed with MA and migralepsy or migraine aura-triggered seizure.

After 1 year, control EEGs subsequently showed rare occipital spike-wave anomalies predominantly right or bilateral.

Since starting treatment, the girl has no longer had seizures or headaches, but only recurrent aura episodes without migraine.

At the age of 13, she started having migraine attacks with aura with sporadic frequency again, she had no further seizures and her last EEG was normal.

### Patient 2

A 6-year-old girl was born premature (gestational age of 27 weeks), suffering from MA and MO since the age of 5 according to ICHD-3 criteria (5). The headache had migraine characteristics with throbbing and severe pain associated with photophobia, phonophobia, and nausea. Rarely, her migraine attacks were preceded by a visual aura characterized by the vision of a colored and scintillating cube. The frequency of the attacks was about two for a month.

She was admitted at the age of 6 to our hospital for a first epileptic seizure characterized by a visual phenomenon, with vision of a colored cube, together with confusion, dizziness, and malaise, followed by loss of consciousness, deviation of the eyes to the right, and stiffening of the limbs. The episode lasted about 20 min and was followed by a migraine attack.

The interictal-EEG performed during the hospitalization showed left posterior slow electrical activity, while brain MRI showed no pathological sign. She was discharged with a diagnosis of idiopathic occipital epilepsy and endorectal diazepam therapy as needed.

Over the next 2 months, she had two more episodes with the same visual and epileptics features; one of the episodes lasted 30 min during which diazepam e.r. and ibuprofen were administered with no effect.

All the episodes were followed by a migraine attack. Control interictal EEGs showed epileptiform potentials with high-voltage spikes are observed in the right temporo occipital region and sporadic slow waves in the left temporo occipital region. The girl was then discharged and diagnosed with migralepsy or migraine aura-triggered seizure.

The patient subsequently presented two other prolonged seizures with the same characteristics, so she was treated with valproic acid 600 mg per day (20 mg/kg/day), then replaced for side effects and poor efficacy with topiramate at a dose of 75 mg per day (2 mg/kg/day). With topiramate, the girl began to have fewer migraine attacks with aura and no longer had seizures.

### Patient 3

A 12-year-old girl was admitted in our hospital for a first epileptic episode.

Until the age of 8 she suffered from MO and MA according to the ICH-3 criteria. Her visual aura was characterized by the vision of “pulsating spots” (scintillating scotoma). The episodes were usually followed by headache with migraine features such as photo- and phonophobia and vomiting.

The first epileptic episode was characterized by her typical migraine aura (vision of a scintillating scotoma) in the left visual half field, progressive loss of consciousness and posture with semi-flexed limbs, followed, after 15 min, by deviation of the head and gaze to the left, and subsequent evolution in tonic-clonic seizure. The crisis subsided at the emergency department with the administration of 2 mg of intravenous midazolam. In the postictal phase, blurring of vision followed until the next day.

Brain MRI was normal. Interictal-EEG showed epileptic alterations with right occipital spikes. She was discharged with diagnosis of probable migraine aura-triggered seizure and with oromucosal midazolam as needed. After 4 years, at the age of 16, she presented a further episode characterized by a scintillating circle-shaped scotoma visualized in the left half field without headache. She then presented progressive loss of consciousness with staring and tonic-clonic seizure. The scotoma phase was quite long (10–15 min), with a short seizure (2–3 min), followed by severe drowsiness and headache that lasted for about an hour. An EEG was then performed, which was normal.

In later years, the patient experienced further migraine attacks with and without aura or, possibly, isolated aura episodes, without having seizures anymore.

## Discussion

In this case series, we present three patients affected by migraine aura-triggered seizure, also known as migralepsy. We consider these cases representative of this type of manifestation for the following reasons: (1) a history of migraine with typical aura that precedes the onset of epilepsy, (2) the same visual symptoms that have always characterized the migraine aura, also induce epileptic seizures, and (3) a recurring association of the phenomena and the ability of antiepileptic therapy to completely control epilepsy while only partially controlling migraine and/or aura.

Migraine and epilepsy are both relatively frequent paroxysmal neurological disorders which can therefore often coexist and their association could thus be attributed to coincidence. However, these conditions also seem to share pathogenic mechanisms such as increased neuronal excitation and ion channels dysfunction (3). Although the possibility of a closer relationship between these two conditions in both clinical and pathophysiological terms has always been a hot topic, there is still no real consensus for the definition of this relationship (8).

In particular, there are two open issues: (1) the need of a more specific/accurate definition of migralepsy, and (2) the boundary between MA and epilepsy with visual symptoms (e.g., occipital epilepsy).

The term “migralepsy” literally derives from the combination of the words migraine and epilepsy and the definition was introduced by Lennox and Lennox in 1960 to describe a disorder in which “ophthalmic migraine (now called MA) with nausea and vomiting is followed by characteristic features of epilepsy” (9). The term “migralepsy” was originally used to define the condition of visual symptoms followed by migraine and subsequently by an epileptic seizure. However, this term may be applied both to an epileptic phenomenon resembling migraine and to a real migraine attack followed by a seizure (10). Therefore, it is a term requiring a better and consensual definition. In fact, migralepsy is described in the headache classification (ICHD-3), but not in the classification of the epilepsies [International Classification of Epileptic Seizures of the League Against Epilepsy (ILAE)] (8–10). Several cases have been presented with the diagnosis of migralepsy or possible migralepsy (6, 7). However, some of them appeared to be occipital seizures (7), or possible ictal focal visual manifestations followed by a generalized tonic–clonic seizure (6). Sances et al. (6) reviewed 50 cases reported in literature and only two cases presented features supporting the diagnosis of migralepsy. Noteworthy, in none of the described cases was an ictal EEG recording available which included the onset of the seizure. Furthermore, it is also important to point out that the term “migralepsy” does not clearly refer to a seizure, that begins with a migraine aura, or to an actual migraine attack followed by (and/or possibly triggering) a seizure, thus, not indicating a specific reference to one of these two clinical entities (10). In view of all this, in our opinion until a consensus is reached to define its meaning, the term “migralepsy” should be replaced by the more appropriate definition of “migraine aura-triggered seizure.” In the ICHD-3, the term “migraine aura-triggered seizure” is proposed for a condition in which an epileptic seizure occurs “during, or within 1 h after, an attack of migraine with aura” in a patient suffering from MA. However, it should be proven that the seizure is really “triggered” by migraine, the association may be in fact be due to the activation of putatively similar mechanisms, or simply be a random association. A triggering effect of migraine, rather than an occasional association, is suggested when a history of epilepsy is lacking or in cases of repeated occurrence (10).

Another important point is the distinction between a migraine visual aura and an epileptic seizure presenting with visual symptoms. Visual auras can occur in migraine or in focal epilepsy (11). The diagnosis is usually based on clinical elements, but this might be challenging in isolated auras. Besides the ICHD-3 (8), the Visual Aura Rating Scale (VARS) provides parameters to facilitate the clinical identification of migraine auras (12). It includes a visual aura duration of 5–60 min (three points) and gradual temporal development ≥5 min (two points), the presence of scotomas (two points) and fortifications (two points), and the homonymous character of the visual sensations (one point). A total score of at least five points is supposed to identify migraine with visual aura with a sensitivity of 91–96% and a specificity of 96–98%. Visual Aura Rating Scale might be helpful in patients presenting with typical migraine or epilepsy, but it can lead to misdiagnosis especially in cases of isolated auras (12). In Table 1, we summarized the characteristics of our patients, including the VARS parameters. To the best of our knowledge, most reported cases of migrainous visual aura followed by epileptic seizures are based merely on the clinical history of the patient or observation. An EEG-video monitoring evaluation proved the epileptic nature of a visual aura, which was previously diagnosed as migraine aura fulfilling the diagnostic criteria for migralepsy (13). Epileptic auras may also be associated with a lack of EEG ictal patterns and in addition to this, most patients with occipital lobe epilepsy lack occipital lobe EEG seizure patterns in non-invasive EEG examinations (13). On the other hand, migraine attacks may be associated with EEG slowing but not with epileptiform discharges. Thus, patients with suspected migraine-triggered epilepsy should be studied carefully with EEG-video monitoring as the clinical findings of visual aura that precede both conditions can be ambiguous (14). Unfortunately, ICDH-3 diagnostic criteria do not include EEG evaluation (8).

A final point of discussion is the definition of the ictal epileptic headache (IEH) as defined in the ICHD-3 (5). Ictal epileptic headache is defined as a headache caused by and occurring during a partial epileptic seizure, ipsilateral to the epileptic discharge, and remitting immediately or soon after the seizure has terminated. Ictal epileptic headache may be followed by other epileptic manifestations (motor, sensory, or autonomic). This condition should be differentiated from “pure” or “isolated” IEH occurring as the sole epileptic manifestation and requiring differential diagnosis from other headache types. Finally, “hemicrania epileptica” is a very rare variant of IEH characterized by ipsilateral location of headache and ictal EEG paroxysms (5). In the previously reported cases of migralepsy or IEH, no specific EEG picture was detected: (a) high voltage, rhythmic, 11–12 Hz activity with intermingled spikes over the right temporo-occipital regions; (b) high voltage theta activity intermingled with sharp waves over occipital region; and (c) bilateral continuous spike- and slow-wave discharges (15).

More recently Parisi et al. have proposed to include as main IEH criterion the ictal EEG and clinical response to antiepileptic intravenous administration. In the absence of this last data, IEH should be classified as “probable IEH” diagnosis (16). The definition of IEH should be used to classify the events in which headache represents the only ictal epileptic feature (15) while the term “hemicrania epileptica” should be maintained for all cases in which an IEH “coexist” and is associated synchronously or sequentially with other ictal sensory-motor events (15).

The same authors supported the theory that the concept of migralepsy is potentially confusing and should not be used to describe the sequence of migraine aura seizure and an ictal EEG recording is mandatory to confirm diagnosis (15, 17).

## Conclusion

The term migralepsy should be replaced by migraine aura-triggered seizure. However, the criteria for diagnosis should be specified better. In particular, the recording of the visual phenomenon with an EEG should be considered a major diagnostic criterion. However, considering that the episodes can be very rare, thus, difficult to be recorded by EEG, some clinical elements should be necessary for the diagnosis: (1) previous history of MA before the onset of epilepsy, (2) the visual aura triggering the epileptic seizure must have the same clinical features as the migraine visual auras not followed by epileptic manifestations, (3) there must be a recurrence in the association between the visual aura and the epileptic fit, and (4) visual symptoms should meet the VARS criteria. In our cohort of patients, all the aforementioned criteria are met at the same time, and we think that this may be congruous with the diagnosis of “migraine aura-triggered seizure.”

## Data Availability Statement

The original contributions presented in the study are included in the article/supplementary material, further inquiries can be directed to the corresponding author/s.

## Ethics Statement

Written informed consent was obtained from the individual(s) and/or minor(s)' legal guardian/next of kin for the publication of any potentially identifiable images or data included in this article.

## Author Contributions

GS, MB, and CR contributed to the writing of the paper. RM, MF, FU, and ST collected the cases. MV conceptualized the study. FV and LP provided supervision. LP wrote the paper. All authors contributed to the article and approved the submitted version.

## Funding

Funding for this research was provided by the Italian Ministry of Health: code 202101_RF_PAPETTI.

## Conflict of Interest

The authors declare that the research was conducted in the absence of any commercial or financial relationships that could be construed as a potential conflict of interest.

## Publisher's Note

All claims expressed in this article are solely those of the authors and do not necessarily represent those of their affiliated organizations, or those of the publisher, the editors and the reviewers. Any product that may be evaluated in this article, or claim that may be made by its manufacturer, is not guaranteed or endorsed by the publisher.
